# Pharmacogenomics in type 2 diabetes management: towards personalized medicine

**DOI:** 10.1186/1479-5876-10-S2-A19

**Published:** 2012-10-17

**Authors:** Cheng Hu

**Affiliations:** 1 Shanghai Diabetes Institute, Department of Endocrinology and Metabolism, Shanghai Clinical Center for Diabetes, Shanghai Key Laboratory for Diabetes, Shanghai Jiao Tong University Affiliated Sixth People’s Hospital, Shanghai, China

## 

There is inter-individual variability in the responses to anti-diabetic treatments, partly due to genetic factors involved in drug absorption, distribution, metabolism and target. The identification of genetic markers related to drug reaction can help physicians with the decisions of drug selection, dose titration, treatment duration, and avoidance of advert drug reactions. We focused on the effects of susceptibility genes for T2D on anti-diabetic drugs’ efficacy. With respect to repaglinide, genetic variants at multiple loci such as *CYP2C8*, *SLCO1B1*, *KCNJ11*, *TCF7L2* and *SLC30A8*, affect either its pharmacokinetics or pharmacodynamics. We also made some efforts on pharmacogenetic studies of repaglinide efficacy. We recruited a total of 104 Chinese patients with type 2 diabetes and with no history of prior antidiabetic medications, to whom subsequent repaglinide monotherapy with a 48-week follow-up was applied. Based on studies on this cohort, genetic variations at *KCNJ11*, *ABCC8*, *NOS1AP* and *KCNQ1* were found to be associated with repaglinide efficacy. Moreover, we also focused on investigations into possible genetic factors for rosiglitazone efficacy, and have already suggested effects of *ABCA1* and *SLC30A8* variants on the response to rosiglitazone treatment. In spite of all these advances in the field of pharmacogenetics of type 2 diabetes, the pace of clinical application of these findings is rather slow. Consequently, more researches especially randomized clinical trials into the practical utility should be conducted.

